# Nanozymes—Hitting the Biosensing “Target”

**DOI:** 10.3390/s21155201

**Published:** 2021-07-31

**Authors:** Yingfen Wu, Diane C. Darland, Julia Xiaojun Zhao

**Affiliations:** 1 Department of Chemistry, University of North Dakota, Grand Forks, ND 58202, USA; yingfen.wu@und.edu; 2 Department of Biology, University of North Dakota, Grand Forks, ND 58202, USA

**Keywords:** nanozyme, biosensing, catalytic activity

## Abstract

Nanozymes are a class of artificial enzymes that have dimensions in the nanometer range and can be composed of simple metal and metal oxide nanoparticles, metal nanoclusters, dots (both quantum and carbon), nanotubes, nanowires, or multiple metal-organic frameworks (MOFs). They exhibit excellent catalytic activities with low cost, high operational robustness, and a stable shelf-life. More importantly, they are amenable to modifications that can change their surface structures and increase the range of their applications. There are three main classes of nanozymes including the peroxidase-like, the oxidase-like, and the antioxidant nanozymes. Each of these classes catalyzes a specific group of reactions. With the development of nanoscience and nanotechnology, the variety of applications for nanozymes in diverse fields has expanded dramatically, with the most popular applications in biosensing. Nanozyme-based novel biosensors have been designed to detect ions, small molecules, nucleic acids, proteins, and cancer cells. The current review focuses on the catalytic mechanism of nanozymes, their application in biosensing, and the identification of future directions for the field.

## 1. Introduction

Enzymes are biocatalysts that accelerate the chemical reactions of many metabolic processes in cells. While they are generally globular proteins, and a few contain nucleic acids, they tend to act alone or in larger functional complexes. The catalytic activity of the enzyme is generally determined by the structure which is specified by the primary amino acid sequence. Usually, the size of the enzyme is larger than the substrate(s), which typically binds in specific active sites determined by the primary, secondary and tertiary structure of proteins in the complex [1]. Enzymes are generally specific to their substrates and reduce the activation energy required to complete the reaction. Because of the properties inherent in the primary, secondary, tertiary, and quaternary structures, enzymes are limited to functional ranges in temperature, pH, and salinity compared with industrial catalysts such as ethylene oxide [2]. Despite their functional range limitations, enzymes have tremendous application potential as biocatalysts. Several technologies have been able to address many structural and functional shortcomings of enzymes such as low operational stability, sensitivity to operational environments, high cost of production, purification quality consistency, and cycling optimization. However, many challenges remain with regard to the effective utilization of biocatalysts in biosensing, including simultaneous discrimination of multiple targets. The combination of biocatalysts with nanotechnology offers an opportunity to address these challenges effectively.

With recent, rapid developments in nanotechnology, nanozymes have attracted significant interest due to their novel and starkly distinct potential when compared with their bulkier, amino acid-based counterparts. Nanozymes are nanomaterials that display enzyme-like properties and can catalyze reactions [3]. These include nanomaterials such as simple metal and metal oxide nanoparticles [4,5], metal nanoclusters [6], quantum dots and carbon dots [7,8], nanotubes, and nanowires [9,10], as well as metal-organic frameworks (MOFs) [11]. These diverse nanomaterials can exhibit catalytic capabilities similar to enzymes but can overcome many of the effective range and stability limitations associated with enzymes. The advantageous features of nanozymes include low cost of production, high catalytic activity, high operational robustness, long shelf-life, and ease of generating modifications [3,12]. Because of their inherent properties, nanozymes can also work as recognition receptors [13] or signal tags [14]. Furthermore, they can be used as signal amplifiers via the utilization of different detection methods such as electrochemistry [15], fluorescence [16], colorimetry [17], immunoassay [18], and other analysis approaches [19]. Nanozymes have recently been utilized in a broad array of applications including biosensing [20], environmental protection [21], antibacterial application [22], cancer therapy [23], and cryoprotection [24].

**Figure 1:**
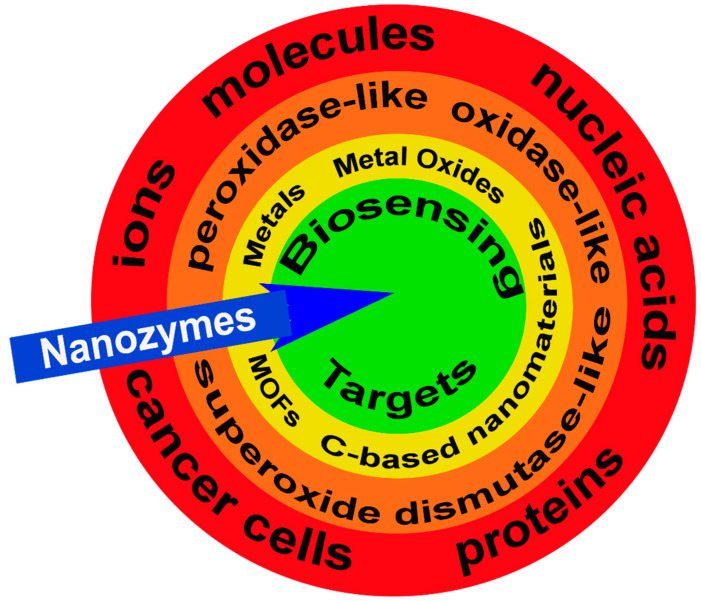
Nanozymes and biosensing targets. Examples of potential targets for nanozymes (blue arrow) including ions, molecules, nucleic acids, proteins, and cancer cells (red band). There are three categories of nanozymes grouped by their functional enzyme-mimicking capacity including, peroxidase-like, oxidase-like, and superoxide dismutase-like (orange band) with the active sites generated by metals, metal oxides, metal-organic frameworks (MOFs), and carbon (C)-based nanomaterials (yellow band). Collectively, these features attract and facilitate the enzymatic reaction at the nanozyme target biosensing target (green circle).

The primary goals of this review are to (1) describe the different types of nanozymes and their functional elements; (2) define their catalytic mechanisms, including limitations, and (3) identify current and future applications for biosensing from ions to tissues (Figure 1).

## 2. Nanozyme Classification and Their Catalytic Mechanisms

Currently, more than 40 types of nanozymes have been reported. All of them embody the same basic framework; they are made of nanomaterials with specific nanostructures able to catalyze biochemical reactions of specific substrates, although the mechanisms are not necessarily comparable to natural enzymes. Nanozymes also show similar enzymatic kinetics and catalytic mechanisms comparable to those displayed by natural enzymes. Based on the activities they exhibit, nanozymes are categorized into two large families: the oxidoreductase family and the hydrolase family. The members of the oxidoreductase family are involved in redox catalysis and function similarly to catalase, superoxide dismutase, oxidase, peroxidase, or nitrate reductase. Members of the hydrolase family are involved in catalyzing hydrolysis reactions in a fashion similar to phosphatase, protease, nuclease, esterase, or silicatein [25]. The major groups are further subdivided below based on the active component of the nanozyme and a comprehensive list of groups, their mechanisms of action, major targets, and detection mechanisms is provided in Table 1 at the end of Section 3.5.

### 2.1. Nanozyme Active Component

#### 2.1.1. Metal Elements

Many metals have enzymatic activity, primarily based on their atomic structure and valence properties, that promote the generation of reactive oxygen species (ROS) and facilitate the electron-transfer process. Noble metals such as gold (Aurum, Au), silver (Argentum, Ag), bismuth (Bi), palladium (Pd), and platinum (Pt) display unique plasmonic features at the nanoscale level, one of which is their large optical enhancement. This is also referred to as the surface plasmon resonance (SPR) [26], which is a phenomenon of plasma resonance resulting in radiant light emission, caused by the resonant oscillation of the free electrons in the presence of light. As a result, for example, Au nanoparticles (NPs) have been designed and applied to different fields, including biosensing, dark-field imaging, and nanomedicine [27,28,29]. Au NPs (positively charged) have been effectively utilized as natural peroxidase mimics for detection of hydrogen peroxide (H_2_O_2_) and glucose in the presence of 3,3′,5,5′-tetramethylbenzidine (TMB) [30]. For example, over a broad dynamic pH range, a folic acid graphene oxide-Au nanocluster hybrid (GFA) has been used to conduct quantitative colorimetric detection of folate receptors on human cervical (HeLa) and breast cancer (MCF-7) cells [31]. The mechanism involves the catalyzation of TMB and H_2_O_2_ by GFA based on its enzyme mimicking activity [31]. Further, Au NPs have been used to detect target DNA or microRNA (miRNA) using complementary nucleic acids immobilized on Au NPs which then facilitated hybridization of the nucleic acids [32,33]. Au NPs also have been used to detect ions and cancer cells [34,35] further supporting the value of this nanozyme in biological applications.

#### 2.1.2. Metal Oxides

The catalytic activities of the metal oxides are comparable to those of the metal ions, except the noble metal elements. The metal oxides in NPs will work as metal ions when combined with H_2_O_2_ because of their different valence values. 

##### Fe Oxides Nanozymes

**Figure 2:**
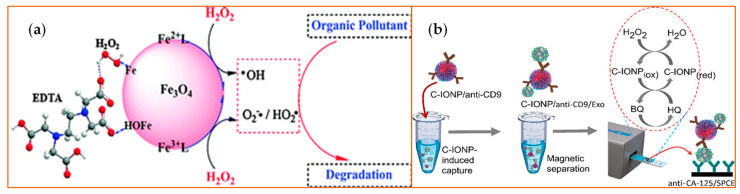
Examples of iron (Fe)-based metal oxide nanozymes. The figure depicts catalysis reactions of H_2_O_2_ decomposition reaction with Fe_3_O_4_ or Fe_2_O_3_ [40] NPs. (**a**) The Fe_3_O_4_ NPs bear active H_2_O_2_ on their surface that generate ROS and, therefore, increase the degradation rates of organic pollutants such as pentachlorophenol, sulfamonomethoxine, and Rhodamine B (RhB). EDTA: Ethylenediaminetetraacetic acid. Reprinted with permission from ref. [41]. Copyright 2010 Royal Society of Chemistry; (**b**) The peroxidase-mimicking activity of the carboxyl group-functionalized iron oxide nanoparticles (C-IONPs) displayed the ability to catalyze the oxidation of TMB in the presence of H_2_O_2_ for the direct isolation and quantification of disease-specific exosomes, as the authors demonstrated using exosomes bearing the ovarian cancer biomarker (CA-125). Exo: exosomes; SPCEs: screen-printed carbon electrodes; HQ: hydroquinone; BQ: benzoquinone.CD9: tetraspainin-9; CA-125: cancer antigen 125. Reprinted with permission from ref. [40]. Copyright 2021 American Chemical Society.

The nanomaterials with iron ions work as peroxidase mimics, generally functioning via a Fenton reaction as advanced oxidation processes (AOPs). Iron oxide-based nanoparticles, including the ferromagnetic (Fe_3_O_4_) NPs, and hematite (Fe_2_O_3_) NPs (Figure 2) have a variety of applications based on the partner with which they are combined. They work as a peroxidase when combined with H_2_O_2_, or function as an oxidase when serving as a glucose sensor. Further, they act as a dual biocatalyst when utilizing a pH-dependent mechanism to display peroxidase and catalase functional potential. Figure 2 illustrates the use of ferromagnetic and hematite products in combination with H_2_O_2_ to target organic pollutants for degradation (Figure 2a) or to detect exosomes (Figure 2b). The fundamental reaction series is depicted in Equations (1)–(7) [36]. The H_2_O_2_ combines with ferrous (Fe^2+^) ions to generate hydroxyl radicals via a complex reaction sequence. Ferric (Fe^3+^) ions also react with H_2_O_2_. This reaction has many advantages such as a low level of iron ion leaching, the efficient cycling of iron ions, low iron sludge production, the wide working pH range, and the reusability as well as the long-term stability of the catalysts [36]. In this way, the iron oxide-based nanomaterials react with or catalyze H_2_O_2_ and can be applied in H_2_O_2_-based reactions. Additionally, the sulfide analogs of magnetite, like greigite (Fe_3_S_4_), which shows the same inverse spinel structure as its oxide counterpart Fe_3_O_4_, exhibits peroxidase-like activity, similar to Fe_3_O_4_ [37]. Based on the different valence states, the other Fe-containing nanoparticles like ferric hexacyanoferrate, Prussian Blue (PB) [38], or magnetic nanoparticles [39] show similar enzyme-like properties.

(1) $${\mathrm{Fe}}^{2+}+H_{2}O_{2}\;\to \;{\mathrm{Fe}}^{3+}+\mathrm{HO}+\mathrm{OH}⊖$$

(2) $${\mathrm{Fe}}^{3+}+H_{2}O_{2}\;\to \;{\mathrm{Fe}}^{2+}+{\mathrm{HO}}_{2}+H⊕$$

H_2_O_2_ + HO → HO_2_· + H_2_O (3)

(4) $${\mathrm{HO}}_{2}\;\to \;{O_{2}\cdot }^{-}+H⊕$$

(5) $${\mathrm{Fe}}^{3+}+{\mathrm{HO}}_{2}\;\to \;{\mathrm{Fe}}^{2+}+O_{2}+H⊕$$

Fe^3+^ + O_2_·^−^ → Fe^2+^ + O_2_ (6)

(7) $${\mathrm{Fe}}^{2+}+\mathrm{HO}+H⊕\;\to \;{\mathrm{Fe}}^{3+}+{\mathrm{HO}}_{2}$$

##### Other Metal Oxides-Based Nanozymes

**Figure 3:**
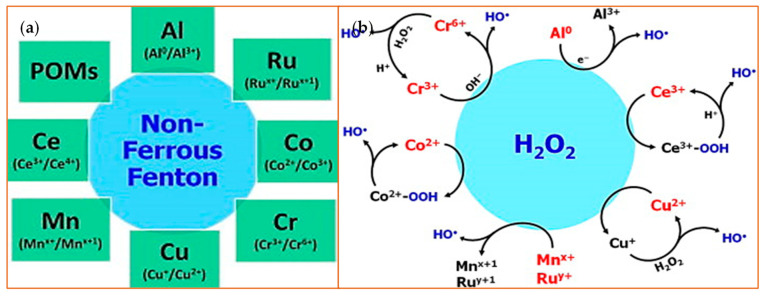
Non-ferrous Fenton ions and their reaction with hydrogen peroxide. (**a**) A schematic is shown that highlights non-Ferrous Fenton ions with the oxidation states that catalyze the substrates [47]. Polyoxometalates (POMs) are metal oxyanion clusters; (**b**) This schematic depicts an overview of the redox reactions between H_2_O_2_ and various non-ferrous Fenton catalysts. The species highlighted in red or blue indicate the active Fenton catalyst and the product, respectively. Reprinted with permission from ref. [47]. Copyright 2014 Elsevier.

Due to their valence properties and outer orbital ring structure, some ions, particularly the transition metals, have multiple oxidation states that gives them the ability to generate the Fenton-like reaction. Therefore, a specific oxidation state of an ion can be regenerated from an inactive state through a simple redox cycle. In this way, many metal ions such as chromium (Cr) [42], cobalt (Co) [43], copper (Cu) [44], manganese (Mn) [45], and ruthenium (Ru) [46], to name a few, react with H_2_O_2_ in a Fenton-like way (Figure 3), with the end result of catalysis of H_2_O_2_. 

##### Mn Oxide Nanozymes

Manganese exists in various oxidation states ranging from 0 to +7; however, only the oxidation states of +2 to +4 have catalytic significance [48]. The reason is that only Mn^2+^ and Mn^4+^ are stable in the aquatic environment which is critical for bioapplications. The facile interconversion between Mn^2+^ and Mn^4+^ via Mn^3+^ ensures that the process of Mn-catalyzed Fenton-like activation of H_2_O_2_ is rapid and efficient. Usually, all Mn ions occur as oxide polymorphs (MnO, Mn_3_O_4_, MnOOH, and MnO_2_), and when they are “doped” or incorporated into NPs they react efficiently with H_2_O_2_ [49,50,51,52]. However, there are caveats associated with the different oxidation states for Mn in that physical form, since chemical composition and concentration can generate different ROS, including HO· and superoxide (O_2_^−^) which are highly cytotoxic.

##### Cu Oxide Nanozymes

**Figure 4:**
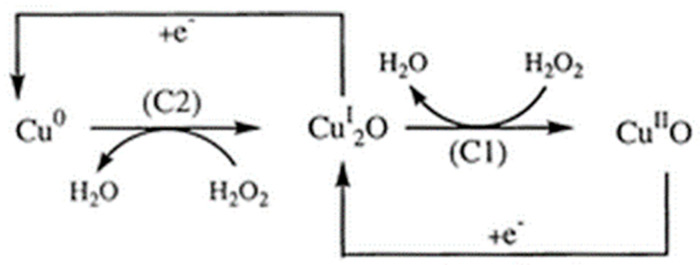
The copper (Cu)-mediated reduction process of H_2_O_2_ at the CuSPE (copper-plated screen-printed carbon electrode) [55]. The transition from Cu^0^ to the Cu^I^_2_O (**left**) and Cu^I^_2_O to the Cu^II^O (**right**) drives the production of H_2_O from H_2_O_2_ with C2 and C1, representing the energy differences at the two cathodes, respectively. Reprinted with permission from ref. [56]. Copyright 2000 Royal Society of Chemistry.

Another potent nanozyme involves Cu ions when combined with H_2_O_2_, which shows redox properties similar to iron. Cu ions have two oxidation states, Cu^2+^ and Cu^+^, both of which can react with H_2_O_2_ easily, similar to how Fe^3+^ and Fe^2+^ react with H_2_O_2_ [53,54]. The difference lies in the fact that in acidic and near-neutral conditions, Cu^+^ reacts with H^+^ and generates Cu^2+^, which reduces the effective Cu^+^ available to react with H_2_O_2_. As a result, the pH value of the solution needs to be corrected before the nanozyme “sensing” process starts (Figure 4).

#### 2.1.3. Metal-Organic Frameworks (MOFs)

**Figure 5:**
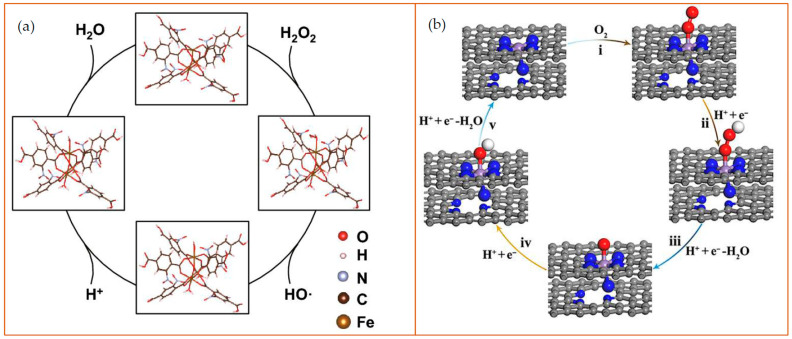
Examples of the catalytic mechanism of MOFs. (**a**) Schematic diagram of peroxidase-like reaction of NO_2_-MIL-101. in an acidic environment. In the MOFs, the Fe was employed at the reaction site to cleave H_2_O_2_ into an ^•^OH and a hydroxyl group (–OH). The hydrogen ions and –OH form H_2_O as a byproduct. Reprinted from ref. [65]; (**b**) Schematic diagram of oxidase-like nanozyme: carbon nanoframe–confined axial N-coordinated single-atom Fe (FeN5 SA/CNF). The pathways of O_2_ reduction to H_2_O is a four-electron process on the nanozyme surface: (i) O_2_ + H^+^ + e^−^ = OOH; (ii) OOH + H^+^ + e^−^ = O + H_2_O; (iii) O + H^+^ + e^−^ = OH; (iv) OH + H^+^ + e^−^ = H_2_O. The color of the dots represent as follows: gray: C; blue: N; purple: Fe; red: O; white: H. Reprinted from [66].

Metal-organic frameworks are a type of nanomaterial that consists of metal ions or clusters of ions connected by organic linker groups. They are crystalline solids that are constructed by self-assembly of single metal cations or metal clusters with organic ligands that possess multiple binding sites [56,57]. Because of the specific shapes, MOFs can specifically and selectively recognize target substrates through Van Der Waals interactions of the framework surface with the substrate, metal-substrate interactions, and hydrogen bonding of the framework surface with the metal ion surface [58]. MOFs have been studied for their rich structural chemistry and potential applications, including biosensing. Their structure contains aromatic or conjugated π moieties (in a molecular system where p orbitals connect with delocalized electrons), which gives them enhanced optical properties [59]. In addition, the metal components also contribute to the increased MOF’s optical properties, for example, lanthanide-based MOFs possess substantial photoluminescence (PL) potential [60]. MOFs are quite promising because of their structural diversity and their tunable chemical and physical properties. The unique chemistry structures have led to their function as effective glucose detectors [61,62]. They have also been used to detect other molecules such as thiamine and cysteine [63], as well as H_2_O_2_ or sulfhydryl-containing compounds. Two examples of the general catalytic mechanisms of MOFs are provided in Figure 5. For the most part, MOFs use functional pores to detect their substrates, which limits their application in biosensing because of the lack of available molecular recognition elements. To address this challenge, recognition capacity has been enhanced by adding open metal sites and specific sites on the pore surfaces, such as with the addition of the zirconium (Zr) ion site on MOFs that can promote the catalysis of H_2_O_2_ due to the high concentration of available Zr-OH catalytic sites [64].

#### 2.1.4. Carbon-Based Nanomaterials

**Figure 6:**
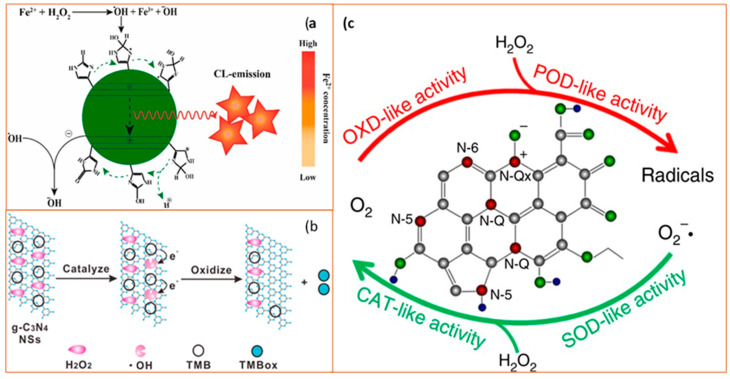
Schematic diagram of catalytic mechanisms of carbon-based nanozymes. (**a**) Schematic illustration of the process of N-CDs enhanced Fenton system which was used for the sensitive and selective determination of Fe^2+^ ion (CL as chemiluminescence). Reprinted with permission from ref. [74]. Copyright 2019 Elsevier; (**b**) Catalytic mechanism of the g-C_3_N_4_ nanosheets (NSs)-H_2_O_2_-TMB system. From left to right: H_2_O_2_ molecules interact with g-C_3_N_4_ NS to generate ^•^OH and ^•^OH oxidize TMB to form a blue product TMBox. Reprinted with permission from ref. [78]. Copyright 2017 American Chemical Society; (**c**) Schematic diagram of enzyme-like activities of N-doped porous carbon nanospheres (N-PCNSs). N-PCNSs perform four enzyme-mimicking activities: oxidase (OXD), peroxidase (POD), catalase (CAT), and superoxide dismutase (SOD) for ROS regulation. Reprinted from ref. [79].

Carbon-based nanomaterials (CNMs), generally have superoxide dismutase-like and peroxidase-like activities and include fullerenes, carbon nanotubes (CNTs), graphene, graphene quantum dots (GQDs), and carbon quantum dots (CQDs) [67,68]. They display excellent physical and chemical properties, high operational stability, and low cost compared with natural enzymes. The unique properties associated with CNMs rely on the fact that carbon is one of the few chemical elements with the ability to polymerize at the atomic level to form long carbon chains as the four electrons in the outer layer can form single, double, or triple bonds with other elements. Moreover, CNMs maintain robustness even in stringent conditions, making them suitable for generating metal-free catalysts [68,69,70]. For example, carboxyl-modified graphene oxide (GO-COOH) has intrinsic peroxidase-like activity when catalyzing the reaction of the peroxidase substrate, TMB, in the presence of H_2_O_2_ to produce a blue-colored reaction product [71]. A series of CNM-based biosensors for H_2_O_2_ [72,73] and other small molecules, ions [74,75], DNA [76,77], protein, and cancer cells have been developed, with the TMB used as a reaction substrate. The addition of TMB provides an added visual signal sensitivity since the TMB is readily oxidized by the carbon-based nanozyme. The general catalytic mechanisms of carbon-based nanozymes for detection of ions and small molecules and the applications based on the mechanisms are provided in Figure 6.

### 2.2. Nanozyme Reaction Mechanisms

Nanozymes were originally designed to overcome the limitations associated with the large-scale, broad application of natural enzymes to maintain a comparable catalytic mechanism relative to the specific substrates and in line with the desired outcome. Below is a summary of the general mechanisms utilized by nanozymes.

#### 2.2.1. Peroxidase-Like Nanozymes

Peroxidase is an enzyme that catalyzes oxidation-reduction reactions using the mechanism of free radical transformation into oxidized or polymerized products [80]. Nanozymes in this group display similar mechanisms when catalyzing the substrates (mostly H_2_O_2_), in which peroxides serve as electron donors. Research reported by Qu et al. has demonstrated that for carbon-based NPs, the functional groups “–C=O” and “–O=CO–” of GQDs can serve as catalytic activity sites and substrate-binding sites, respectively [81]. In contrast, the presence of an “–C–OH” group will inhibit the catalytic property of GQDs. At the mechanistic level, the aromatic domains of CNMs serve as the peroxidase mimic, catalyzing the reaction of H_2_O_2_ to ·OH whereas the –COOH reacts with H_2_O_2_ to generate –C(OH)_2_OOH, and subsequent H_2_O isolation produces –O=C–OOH [82]. In addition to the carbon-based nanozymes, metal oxidases such as Fe_3_O_4_ [4], single atom nanozymes such as Fe-N-C nanozymes [83], and MOFs such as Cu(PDA)(DMF) [84], also serve as peroxidase mimics to form ·OH when the substrate is H_2_O_2_ [4]. Knowing the mechanisms of peroxidase-like nanozymes benefits the design of the project for the catalysis of H_2_O_2_ and H_2_O_2_-based applications, including the biosensing of glucose [85], cysteine [86], and uric acid [87]. With the advancement of nanotechniques, researchers also used the peroxidase-like activity of nanomaterials for the detection of alkaline phosphatase [83], galactose [88], and ions [35]. Efforts related to exploring the potential of peroxidase-like nanozymes not only focused on expanding the primary application but also on improving the catalytic properties of the nanozymes to decrease the impact coming from the reaction conditions. Chen et al. designed negatively charged liposome-boosted, peroxidase-mimicking nanozymes to exhibit the activity in even alkaline conditions [89]. Zhao et al. found that DNA modification made the activity 4.3-times higher compared with that of bare MoS_2_ nanosheets [90] lending further support to the value of using peroxidase-like nanozymes.

#### 2.2.2. Oxidase-Like Nanozymes

An oxidase is an enzyme that catalyzes oxidation-reduction reactions. In biological subjects, the oxidases could be used to catalyze the production of glucose, monoamine [91], and other substrates [92,93]. The oxidase-like nanozymes are designed to mimic the properties of oxidases. The oxidase-like nanozymes can be separated into two groups based largely on their catalytic mechanism: the glucose oxidase-like group and the sulfite oxidase-like group. Noble metals such as Au can form Au^+^-O_2_^−^ or Au^2+^-O_2_^2−^ couples, generating a dioxo-Au intermediate that can serve as a bridge to transfer electrons from glucose and H_2_O_2_ to dioxygen and water in a glucose detection reaction [94]. An alternative mechanism is mediated by the sulfite oxidase-like group in which the sulfite oxidase functions as the electron acceptor during catalysis. For example, molybdenum trioxide (MoO_3_) NPs possess an intrinsic sulfite oxidase-like activity the mechanism of which has been determined: MoO_3_ NPs catalyze the oxidation of colorless 2,2′-azino-bis(3-ethylbenzothiazoline-6-sulfonic acid) (ABTS) to generate a green reaction product. The mechanism was used to detect acid phosphatase (ACP) because the ACP catalyzed the hydrolysis of the ascorbic acid 2-phosphate (AAP) substrate to produce ascorbic acid (AA). As a result, the AA reduces the colorimetric output from ABTS oxidation [95] generating a highly sensitive biosensing tool. 

#### 2.2.3. Superoxide Dismutase-Like Nanoparticles

**Figure 7:**
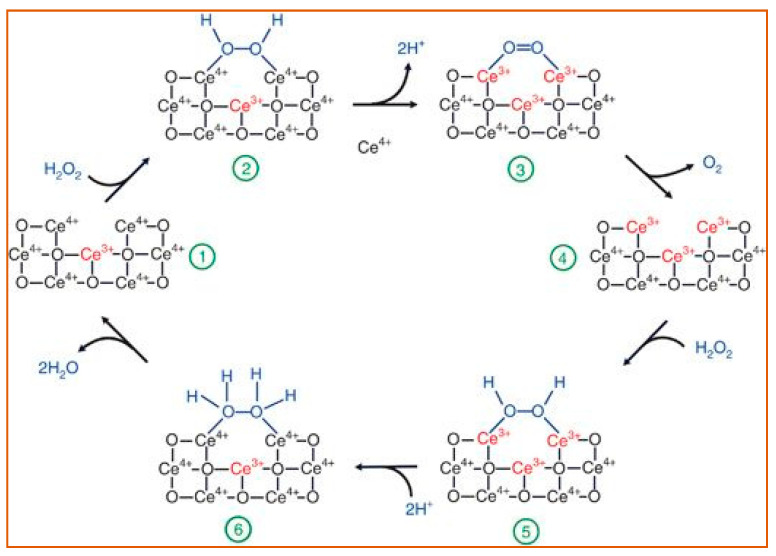
A review of the reaction mechanism of cerium oxide nanoparticles (CeO NP) catalyzes the H_2_O_2_ with its superoxide dismutase-like activity. During the entire process, the amount of bound Ce^3+^/Ce^4+^ (red font) changes with the structure of the oxygen-containing groups (blue font), causing the catalyzation of H_2_O_2_ [97]. The reactants and products are indicated at each stage of the reaction, 1–6. Reprinted with permission from ref. [97]. Copyright 2011 Nanoscale.

Ceria (CeO_2_) NPs are the main members of superoxide dismutase-like nanoparticles. Because of the oxidase states of Ce^3+^ and Ce^4+^, nanoceria may transition between the two in a redox reaction, producing oxygen vacancy sites. Based on the redox capacity, nanoceria is considered an acceptable oxygen buffer [96,97]. This unique electron structure makes CeO_2_ an essential example of a superoxide dismutase-like nanoparticle [98]. The main catalysis process is illustrated in Figure 7.

Overall, nanozymes function as peroxidase, oxidase, superoxide dismutase, or other enzymes based on the characteristics and potential of their active components, including the different charge statuses or the innate properties derived from their structures. Different nanomaterials mimic the activity of different enzymes. The mimic activity can be affected by the structure of nanozymes, including the particle size [99], surface modification [100,101], and morphology [102] to improve the catalytic activity, substrate specificity, and stability [103,104]. Many nanozymes are size-dependent since smaller nanozymes show a higher surface-to-volume ratio, which enhances the interactions with substrates because of the more active sites exposed [99]. But sometimes larger nanozymes may show higher activity, possibly because there are more metal ions [105], the presence of suitable valences [106], or the existence of redox reaction potential [107]. Excellent examples of these include two-dimensional (2D) nanomaterials, with the characteristic single-layer nanosheet structure that can include graphene, hexagonal boron nitride, transition metal dichalcogenides, graphitic carbon nitride, layered metal oxides, or layered double hydroxides. The advantage of these 2D nanomaterials is that they possess high specific surface area, numerous active sites, the ability to act as supporting materials within a larger structure, and show enhanced nanozyme catalysis activity [108,109,110,111]. There is also tremendous potential for surface modification which can include coating with small molecules, ions, and polymers on the surface, thereby increasing the stability and active reaction sites available. These surface modifications, therefore, can be used to adjust or “fine-tune” the catalysis properties of this class of nanozymes [112,113,114]. Additionally, the morphology and crystallographic planes have important effects on modulating the catalytic activity because the different amounts of types of available bond structures and various arrangements of atoms in the nanozymes determine the selectivity and reactivity of nanozyme, overall [115,116]. In addition to the structural composition [117] of nanozymes, their reaction environment, such as pH, temperature, and light are key factors that can affect nanozyme activity [118,119,120]. Determining the relationship between the structure and the catalysis activity will help us to design nanozymes in the future with high activity and specificity. With the development of nanotechnology, more nanozymes will be designed and studied to further advancements in biosensing and related fields. 

## 3. Nanozymes and Their Potential Applications in Biosensing

With the rapid technological advances associated with nanozymes, the broad application of nanozymes extended apace to different fields, including environmental protection [35], anti-bacterial treatment [121], cancer therapy [122], cytoprotection [123], biosensing [101], and more [124]. Advances were achieved using different methodological approaches, including optical (fluorescent [125] and photoluminescent [126]) and electrochemical (voltametric [127] and amperometric [128]) detection strategies. Among these applications, the usage of nanozymes in biosensing has drawn notable attention because of the increased need for stable, cost-effective catalytic tools for use in clinical and basic research. While the classical view of biosensors generally refers to a biological component in combination with a chemical component or partner, we take a more broad view herein to include chemical structures (and/or devices) that can be used to detect a biologically relevant target. The most rapidly expanding area of research and application is in biosensing with the major application approaches highlighted below and summarized in Table 1.

### 3.1. Detection of Ions

Metal ions, especially heavy metals, are not easily metabolized and, therefore, accumulate in organs resulting in tissue damage and increasing disease vulnerability over time [129,130]. As a result, the accurate detection of metal ions in the environment or tissues is urgently needed as a prelude to environmental remediation [131] or clinical intervention strategies [28,132]. Here we give two examples linked to mercury (Hg) and Cu ions to illustrate the critical characteristics of the materials and the proposed mechanism of action for target ion detection.

**Figure 8:**
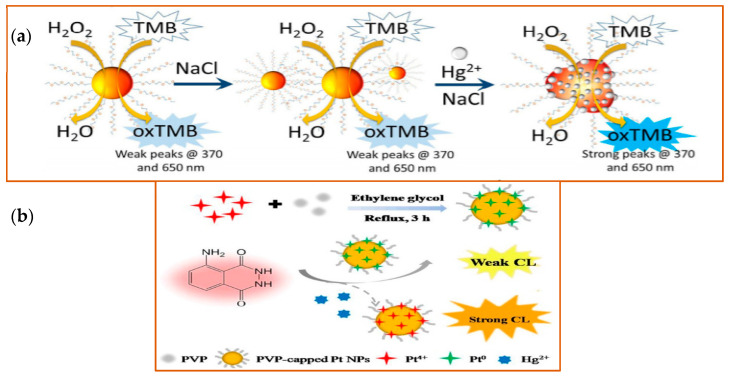
Schematic illustration of detection of Hg^2+^ with different nanozymes. (**a**) Schematic illustration of the detection of Hg^2+^ of Au NPs. Left side, Au NPs show stability in high electrolyte solutions. Right side, in the presence of Hg^2+^, the catalytic activity of the Au NPs was improved, with a strong fluorescence signal detected at the indicated wavelengths. Reprinted from ref. [134]; (**b**) Schematic illustration of Hg^2+^ detection by Pt NPs. Pt NPs capped with PVP (polyvinyl pyrrolidone) were synthesized in the mixture solution of Pt^4+^, PVP, and ethylene glycol under 3 h reflux. With the peroxidase-like activity, PVP-capped Pt NPs catalyzed the CL of the luminol system in the presence of Hg^2+^. Reprinted with permission from ref. [135]. Copyright 2019 John Wiley and Sons.

As an extremely toxic metal, mercury is one of the important targets detected by different nanozymes. Mercury exposure has been linked to brain, kidney, and lung damage and has been identified as a primary cause or contributing factor in several diseases, including Minamata disease, Alzheimer’s disease, cardiovascular disease [133] among many. Cao and his colleagues found that when Au NPs were functionalized with oligo-ethylene glycol (OEG), the formation of an Au-Hg amalgamation was enhanced. Indeed, with this approach, they were able to achieve a low limit of detection (10 ppb) in lab-based water samples (Figure 8a) [134]. Platinum (Pt) nanozymes, based on their peroxidase-like function, can also be used to detect mercury ions because Hg^2+^ was specifically shown to inhibit the catalytic properties of the nanozymes in a luminol system. Zhao et al. found that Pt NPs can catalyze the chemiluminescence (CL) of the luminol system. They took advantage of this catalysis mechanism and applied it toward Hg^2+^ detection, as the Hg^2+^ could further enhance the CL intensity in the Pt NPs-luminol CL system. With this approach, they were able to detect Hg^2+^ and achieved a low-end detection limit of 8.6 nM compared with other methods (LOD ranges from 3.3 nM to 338 nM) (Figure 8b) [135]. Based on the intrinsic properties of Au and Pt, Wang and colleagues designed an Au@AgPt NP with surface-enhanced Raman scattering (SERS)—an active peroxidase-like activity that could be used to detect the signal molecules (which generate SERS or colorimetric signal) [136]. SERS is a sensing technique in which inelastic light scattering by molecules is enhanced when the molecules are adsorbed onto corrugated metal surfaces [137]). With the help of a colorimetric/SERS dual-mode probe integrated with the advantages of facile detection by colorimetric analysis and a high-sensitivity trace assay by SERS, Au@AgPt NPs achieved the limit of detections (LODs) of colorimetric analysis of 0.52 μM and by SERS assay of 0.28 nM. Besides the noble metal examples provided, metal oxides nanomaterials can also be used to detect Hg^2+^ [138].

Copper (Cu) is an important element for biological organisms and the proteins that contain Cu are critical for a variety of physiological processes [139,140]. However, high concentrations of Cu can cause cellular damage as has been demonstrated in Wilson’s disease, an inherited disorder characterized by abnormal accumulation of Cu, predominantly in the liver [141]. Therefore, it is important to perform Cu ion detection with the lowest LOD possible. To address this challenge, a wide range of materials have been used to synthesize nanozymes optimal for sensitive Cu detection. Wang et al. reported a rapid and sensitive fluorescence nitrogen-doped GQDs (N-GQDs), which were utilized as sensing probes for the selective detection of Cu^2+^ by taking advantage of the PL quenching of N-GQDs after adding Cu^2+^ [142]. The detection limit for Cu^2+^ was found to be 57 nM. Yan Liu et al. designed nanozymes with noble elements; Au nanoclusters designed for the detection of Cu^2+^ in blood samples attained a minimum detection limit of 0.1 nM. This lower LOD was achieved by the combination of peroxidase-like nanozyme activity of the Au cluster with the amino acids’ ambidentate of histidine (His) because of the peroxidase-like activity of histidine-Au nanocluster (His-AuNCs) could be decreased by adding Cu ions. Additional methods to detect Cu^2+^ have been developed. Raibaut designed a nanozyme that combined the selectivity and suitable affinity of the amino-terminal Cu^2+^- and Ni^2+^-binding (ATCUN, also called Xxx-Zzz-His peptide motif, Xxx can be any amino acid, Zzz can be any but not proline) with the long-lifetime emission of the lanthanide Tb^3+^ to achieve the selective and reversible detection of Cu^2+^ [143]. These examples highlight the value of using nanozymes to detect Cu ions and similar design approaches can be applied to other biosensing targets. 

### 3.2. Detection of Small Molecules

Hydrogen peroxide (H_2_O_2_), an essential oxidizing agent, is generated during many physiological processes, including the oxidation of glucose, and is harmful to cells because of the high oxidation activity relative to proteins and DNA. The detection of H_2_O_2_ could help clinicians and researchers investigate disease progression and mechanisms, such as detecting early-stage vascular disease [144]. Therefore, different methods have been developed to detect H_2_O_2_ using different nanozymes with a wide range of intrinsic catalytic properties [145]. Since H_2_O_2_ is an oxidation product of glucose in the presence of glucose oxidase, there is a clear link between these critical molecules. In fact, clinical and basic research approaches will often attempt to detect H_2_O_2_ and glucose in parallel using compatible approaches [146]. Liang et al. designed Vanadium oxide (V_2_O_5_)-based nanozymes to detect H_2_O_2_ and glucose because of their peroxidase-like activity in the presence of the enzyme-substrate o-phenylenediamine (OPD) [17]. With this dual approach, (V_2_O_5_)-based nanozymes were able to achieve a minimum detection limit of 1 µM for H_2_O_2_ and 10 µM for glucose. The detection of H_2_O_2_ is conducted by various nanozymes, not limited to metal oxides nanozymes. Noble metal nanoparticles could be used to detect H_2_O_2_ and glucose as well. Wang et al. designed palladium-based nanostructures, PdCuAu NPs, which have excellent catalytic performance as peroxidase-like enzymes [147]. The combination of PdCuAu NPs can catalyze TMB rapidly in the presence of H_2_O_2_ and oxidize it to visible blue products (oxTMB). The LODs were 5 nM and 25 nM for H_2_O_2_ and glucose, respectively. As expected, some MOFs were designed to detect the critical molecules H_2_O_2_ and glucose. In Yuan’s work, Fe-MOFs, using the ferric ion as the metal center, incorporated a porphyrin analog as the organic ligand to work as a metalloenzyme which displays unique catalytic properties. This analytical tool was developed to detect H_2_O_2_ and glucose based on its high peroxidase-like catalytic activity [148]. The detection limits of H_2_O_2_ and glucose were 1.2 μM and 0.6 μM, respectively, using this approach.

Other small molecules have been detected successfully using nanozymes as biosensors. Sharma et al. designed a nanozyme that combined Au nanoparticle (GNPs) with an ssDNA aptamer (Ky2), that shows specific molecular recognition elements for kanamycin and blocked the ability of GNPs to catalyze when bound to the Ky2 aptamer. However, in the presence of kanamycin, the Ky2 transferred to the kanamycin and the GNPs were able to catalyze the colorimetric detection of TMB, generating an on/off switch mechanism that was remarkably effective [20]. Shamsipur et al. synthesized a new colorimetric biosensor for glutathione (GSH) based on its radical restoration ability. The carbon nanodots (CDs), enhance the free radical formation generated by the oxidation of TMB by CDs. The free radical cation concentration was related to the GSH concentration, permitting indirect calculation of GSH concentration with a low LOD: 0.3 μM [149]. With new nanozyme synthesis and design techniques developing at a rapid rate, the ability to detect different molecular targets with greater sensitivity and accuracy is increasing. The investigation of potential biosensing applications and the promising potential of nanozymes is attracting increasing research and clinical attention worldwide.

### 3.3. Detection of Nucleic Acids

The detection of nucleic acids by biosensors has been successful using a variety of strategies and engineered for different applications. For DNA, nanozymes are not just used to detect double-stranded DNA, but also single-strand DNA, mutant DNA (compared to “normal” sequence), as well as DNA modifications, such as methylation. Shen and colleagues designed a DNA-controlled strategy for the growth of Pt NPs on graphene oxide (GO–PtNPs) to detect specific DNA targets [150]. They used two hairpin ssDNA and one triplex-hybridization chain reaction (tHCR) to trigger hybridization with a DNA target to form a long double-stranded DNA structure. This allowed the Pt NPs to grow with the Pt precursor on the surface of GO and generate the TMB-based colorimetric assay reactant. However, if there is no DNA target, then the two short hairpin ssDNA would attach on the surface of GO, and Pt NPs growth on the surface of ssDNA occurred without the colorimetric reaction. The proposed method showed very high sensitivity with the detection limits down to 14.6 pM for mutant Kirsten RAt Sarcoma (KRAS) DNA and 21.7 pM for let-7a microRNA, both of which are frequently mutated in tumors. Yao et al. designed TiO_2_ nanowires (NWs) as an effective sensing platform for rapid fluorescence detection of single- and double-stranded DNA [151]. The fluorescence-labeled DNA probes were effectively absorbed by TiO_2_ NWs, and the leading fluorescence intensity helps with the detection of DNA. Zheng et al. proposed unmodified Au NPs for rapid colorimetric detection of DNA methylation-based on the difference in electrostatic attraction of single-stranded DNA and double-stranded DNA against salt-induced aggregation of Au NPs. The principle is that the methylated P53 fragment maintains the methylation status after the bisulfite treatment and leads to a proper match with a designed ssDNA and subsequent aggregation with the AuNPs. An unmethylated P53 fragment will lead to a mismatch with designed ssDNA and dispersion under the treatment of AuNPs. This method has demonstrated a DNA methylated detection limit of 8.47 nM [77] and highlights this approach as a potential strategy to investigate epigenetic modifications of the chromatin landscape.

For RNA detection, identifying specific miRNAs associated with the disease are particularly important in cancer detection and staging. For example, miR-21, a potential biomarker of oral cancer [152], ovarian cancer [153], and other cancers can now be detected using nanozyme biosensors. An ultrasensitive electrochemical biosensor for miR-21 detection was designed based on a padlock exponential rolling circle amplification (P-ERCA) assay [154] and CoFe_2_O_4_ magnetic nanoparticles (CoFe_2_O_4_ MNPs) [155]. This assay is a highly specific and sensitive amplification method with a detection limit down to the zeptomole level designed with a padlock probe composed of a hybridization sequence to miRNA and a nicking target site for the endonuclease. With this approach, Nan Yu and colleagues achieved a wide dynamic range of 1 fM to 2 nM with a low detection limit of 0.3 fM for miR-21 detection [155]. Also, miRNA-155, an oncogenic miRNA in breast cancer [156], non-small cell lung cancer [157], and other cancers, is detectable using a biosensor combined with a nanoscale copper-based metal-organic framework assembled by Pt NPs and horseradish peroxidase (Cu-NMOF@PtNPs/HRP) and a toehold strand displacement reaction (TSDR, an enzyme-free DNA strand displacement reaction based on the principle of toehold exchange to achieve the DNA amplification [158]) to improve the multiple amplifications [159]. In the presence of miRNA-155, the TSDR system would be triggered, leading to the hybridization of the nanoprobe. With this approach, a minimum detection limit of 0.13 fM miRNA-155 can be achieved in RNA extracts of serum and MCF-7 and MDA-MB-231 cell lysates [99]. As the need for rapid and early detection of cancer biomarkers increases, the nanozyme-based biosensors with the highest sensitivity will be of tremendous value.

### 3.4. Detection of Proteins

Proteins play essential roles in our body and misregulation or misexpression of proteins is often at the heart of a variety of diseases. As a result, changes in protein expression, post-translational modification, or folding can be used as biomarkers or hallmarks of disease or disease stage. Therefore, developing approaches that allow for improved quality and quantity of detection is important for the diagnosis and treatment of diseases. Several nanozyme-based approaches have been designed expressly for the purpose of ultrasensitive biomarker detection. Wang and colleagues designed Au/Co bimetallic nanoparticles decorating a hollow nanopore carbon framework (Au/Co@HNCF) for the detection of uric acid in human serum with the limit of detection at 0.023 μM [160]. Uric acid is a hallmark of gout, tumor lysis syndrome, Type 2 diabetes, and other health problems. MOFs, as promising nanoparticles, may have more potential applications compared to metric nanoparticles based on their structure, potential for surface modifications, and tunability. Li et al. used Fe-MIL-88A, a photoactive ion-based MOF material, to detect thrombin based on the peroxidase-like catalytic activity of Fe-MIL-88A towards TMB [161]. Thrombin is an important serine protease, catalyzing many coagulation-related processes, such as cerebral ischemia and infarction [162]. In the presence of thrombin and its corresponding aptamer, the mimetic activity of Fe-MIL-88A is strongly inhibited and is the basis for colorimetric detection and quantification of thrombin with a low LOD of 10 nM. The advantage of this method is that the thrombin could be changed to other target proteins when applying the corresponding aptamers. An easy and simple method for synthesizing nanozymes and detecting various proteins is advantageous for clinic diagnosis and other fields. Detection of various proteins with nanozymes is a large-scale project with several potential strategies and merits future research focus.

### 3.5. Cancer Cell Detection

The detection of cancer cells in the human body is a promising field for biosensors, as the selectivity and sensitivity of the methods are relevant to clinical diagnosis, treatment, and prognosis of cancers. Tian et al. designed CuO nanozymes as a catalyst for the detection of circulating tumor cells with the support materials of reduced graphene oxide/gold nanoparticles composites (rGO/Au NPs composites). On the rGO/Au NPs composites, the MUC-1 (Mucin 1, a cell surface-associated protein) aptamer was used to recognize the MCF-7 cells because of the over-expression MUC-1 on the surface. The CuO nanozyme is used as a signal-amplifying nanoprobe and achieves a low detection limit of 27 cells per mL^−1^ [53]. Zhao et al. designed a cancer cell detection method that combines Au, whole tobacco mosaic virus (TMV), and folic acid to target folic acid receptors on the surface of HeLa cells and other tumor cells [163]. The authors developed an Au@TMV nanowire (AT) conjugated folic acid (FA) complex (ATF) [163]. Because folate receptors are overexpressed on the surface of Hela and other tumor cells, their receptors could bind to the folic acid in the complex. Subsequently, the peroxidase properties of ATF were used to convey a TMB/H_2_O_2_-based colorimetric method to detect folic-acid expressing cancer cells in a mixed population with a detection limit of 2000 cancer cells/mL could be achieved, which is higher than the other nanoparticles using TMV as the basic material [164,165]. While Au NPs can be used for the detection of cancer cells, there are active areas of research investigating other potential sources for materials, focusing on increased specificity and reduced toxicity. Tuncel and colleagues have developed a rapid colorimetric method to detect tumor cells that utilizes an externalizable complex of monodisperse-porous silica microsphere that contains immobilized Fe_3_O_4_ NPs (Fe_3_O_4_@SiO_2_ microspheres). After combination with hyaluronic acid (HA), a ligand sensitive to CD44 receptors on tumor cells, the nanocomposites could be taken up by human cervical cancer (HeLa) cells and primary brain tumor cells T98 G cells via pinocytosis, with the cell detection achieved by oxidation of TMB generated by the catalytic properties of the Fe_3_O_4_@SiO_2_ microspheres [166]. The detection of cancer cells is achieved by the evaluation of the over-expressed surface biomarkers (e.g., CD44 receptors, folic acid [167]) or the amount of internalization of nanozymes that caused changes in cancer cells. There are more methods under evaluation. A comprehensive table that summarizes the nanozyme classification, major substrates, and detection methods is built as Table 1. 

**Table 1:** Nanozyme classification, major substrates, and detection methods.

Groups	Nanozymes	Targets	Signal	Detection Methods	Ref.
Metal-based nanozymes	Au@Pt nanozyme	Ag^+^	extinction spectra	UV–Vis spectroscopy	[35]
	Au NPs	H_2_O_2_	absorbance	adsorption spectroscopy	[30]
	Au NPs	microRNA	surface plasmon resonance (SPR)	hEC-SPR1010 device	[33]
	Au NPs	cancer cells	absorbance	adsorption spectroscopy	[34]
Metal oxidase-based nanozymes	Fe_2_O_3_ NPs	exosomes	absorbance	adsorption spectroscopy	[40]
	CeO_2_ microspheres	phosphoprotein	absorbance	UV–Vis spectrophotometer	[98]
	Fe_3_O_4_@SiO_2_ microspheres	cancer cells	absorbance	UV–Vis spectrophotometer	[166]
	hollow MnFeO oxide	Hg^2+^	absorbance	UV–Vis spectrophotometer	[138]
	MnO_2_ NPs	H_2_O_2_	fluorescence	confocal laser scanning microscopy	[52]
MOFs	Ni/Cu-MOFs	glucose	current	semiconductor parameter analyzer and four-point probe station	[61]
	Cu-NMOF@PtNPs/HRP	miR-155	square wave voltammetry	electrochemical workstation	[159]
	Ni-hemin MOFs	cancer cells	absorbance	SPECTROD 250-analytikjena spectrophotometer	[167]
Carbon-based nanozymes	N-CDs	Fe^2+^	chemiluminescence	BPCL Luminescence Analyzer	[74]
	C-dots	DNA damage	fluorescence	an Infinite 200 PRO multi-mode reader	[76]
	GO-based nanozyme	homocysteine	absorbance	UV-vis spectrophotometer	[112]
	rGO/Au NPs composites	cancer cells	amperometric signals	CHI660E electrochemical workstation	[53]

## 4. Challenges and Future Directions for Nanozyme Research

Although nanozymes show excellent advantages over the traditional biocatalyst approach, several challenges remain to be overcome. One of the major challenges is the poor selectivity and lower sensitivity of nanozymes compared with natural enzymes. The second challenge lies in the fact that there are a far greater number of enzymes than there are currently available nanozyme mimics. If we could develop general nanozyme design workflows along with increasing the number of nanozymes designed to mimic physiologically and clinically relevant enzymes, the potential applications would be essentially unlimited. The third major challenge is related to toxicity concerns because few of the mechanisms or potential toxicities have been identified to date. This latter challenge will likely remain a major hurdle to overcome in the field of nanozyme application in biosensing and will undoubtedly be an active area of future research. 

Based on these challenges, more investigation is imperative to improve the catalytic activity and specificity for nanozymes, while reducing potential toxicity. With the rapid development of nanotechnology, more nanomaterials are currently being developed for use as biosensors due to their enzyme-like properties and ability to mimic catalase, oxidase, peroxidase, phosphatase, and superoxidase dismutase activity. The catalytic activity is determined by the intrinsic chemical structure. Because of the low sensitivity and selectivity, as well as the catalytic property of nanozymes compared with enzymes, studies focused on improving the catalytic activity have drawn the most attention. For example, nanoparticles capped with DNA have been found to improve the oxidation reaction rate because of the long length and sequence [168]. Liu and Lui found that the iron oxide nanoparticles capped with DNA demonstrated higher peroxidase activity than naked nanoparticles. The catalysis activity was enhanced with longer DNA strands and a higher proportion of cytosine relative to the other nucleotides [168]. Qiu et al. used Tris-(benzyltriazolylmethyl) amine (TBTA) to improve the sensitivity and stability of the sensing system to achieve sensitive detection of Cu [169]. 

Another research area ripe for exploration is the expansion of the type and range of nanozyme targets. For example, the detection of tumor markers could be designed to improve both the diagnosis accuracy and time-to-diagnosis, particularly in point of care treatment [170,171]. If diagnosing tumors or tumor types can be done earlier, and more accurately, the treatment efficacy would be dramatically improved. Additionally, the mechanism of inhibition on the enzymatic activities of nanozymes is an important focus area. Further, in their recent review, Fan and colleagues highlight the importance of research on the factors which impact the catalytic efficiency and detection limits of nanozymes [172]. There is also a considerable opportunity at the level of the properties and potential of nanomaterials, themselves, to explore functional mimics that can be utilized to expand biosensing targets.

Rational design of nanomaterials means “design-for-purpose”, a strategy of designing new nanomaterials based on the ability to predict how the new nanomaterial can affect the target and exhibit the appropriate catalytic function to match the target strategy [173]. The clear insights resulting from the integration of active sites, knowledge of nanozyme mechanisms, the grasp of new nanomaterials, and the ability to fuse these together will help researchers to design a new cadre of nanozymes with high-performance potential [174]. The rational design of nanozymes is of great significance for biosensing, biomedical application, and other fields [175,176,177]. For example, Lew et al. designed an N-doped carbon nanocage with Co-Nx active sites (CoNx-NC), as one of the metal nitrogen-doped carbon (metal-NC) catalysts [178]. This unique nanozyme shows both catalase- and oxidase-like properties to detect acetylcholinesterase without peroxidase-like properties. CoNx-NC decomposed H_2_O_2_ into O_2_, thus oxidizing TMB into a blue reaction product. Based on the inhibitory effect of thiocholine on the TMB color reaction, thiocholine is produced in the presence of acetylcholinesterase, which can be used as an indicator of Alzheimer’s disease. Liu et al. designed an arginine (R)-rich peptide/platinum hybrid colloid nanoparticle cluster to mimic the uricase/catalase system and superoxide dismutase/catalase system to degrade uric acid and eliminate ROS [179]. This approach has potential applications for detecting gout and for use in hyperuricemia therapy. To achieve the defined goal, all the characterization and components of nanozymes need to be designed to serve the specific functions and then investigated thoroughly for efficacy. For example, in the CoNx-NC project, to achieve the detection of acetylcholinesterase, the Co-Co Prussian blue analogs, classic cubic MOFs were used as precursors, while polyvinylpyrrolidone (PVP) was introduced to provide extra nitrogen for doping to support the formation and inhibit aggregation. After pyrolysis and acid etching, the Co-Nx-NC was synthesized and applied for acetylcholinesterase detection with oxidase- and catalase-mimicking properties of Co-Nx-NC. Another example is biomimetic nanozymes for glucose detection that have been designed by Geng et al. [180]. They combined amphiphilic amino acid, a histine derivative for fabricating nanoassemblies with the assistance of metal ions, and a heme derivative, which included iron ions in the center. The side chain of the histine derivative and the iron ion of the heme derivative combined through noncovalent interactions and showed peroxidase mimicking property. In this way, supramolecular peptide nanozymes with peroxidase-like activity were designed and synthesized for glucose sensing.

Collectively, nanozyme research and nanozyme applications in biosensing represent tremendous potential for clinical and research benefits. With an increasing focus on designing nanozymes with increased specificity and reduced toxicity, there is promise that we can hit a broad range of biosensing targets.

## Data Availability

Not applicable.

## Abbreviations

ROS: reactive oxygen species; 5mC: methylated cytosine; AA: ascorbic acid; AAP: ascorbic acid 2-phosphate; ABTS: 2,2′-azino-bis(3-ethylbenzothiazoline-6-sulfonic acid); ACP: acid phosphatase; AFP: alpha fetoprotein; AOPs: advanced oxidation processes; AT: Au@TMV nanowire; ATBTS: colorless 2,2′-azino-bis(3-ethylbenzothiazoline-6-sulfonic acid); ATCUN: amino terminal Cu^2+^- and Ni^2+^-binding; ATF: an Au@TMV nanowire (AT) conjugated folic acids (FA) complex; Au NPs: gold nanoparticles; BMCNTs: bamboo-like magnetic carbon nanotubes; BQ: benzoquinone; CeONP: cerium oxide nanoparticle; C-IONPs: carboxyl group-functionalized iron oxide nanoparticles; CL: chemiluminescence; CNMs: Carbon-based nanomaterials; CNTs: carbon nanotubes; CQDs: carbon quantum dots; CuSPE: copper-plated screen-printed carbon electrode; EDTA: Ethylenediaminetetraacetic acid; EtBr: ethidium bromide; Exo: exosomes; FA: folic acids; FAM: fluorophore; FIA: low injection analysis; FRET: Förster resonance energy transfer; GFA: folic acid graphene oxide-Au nanocluster hybrid; GO-COOH: Carboxyl-modified graphene oxide; GOx: glucose oxidase; GQDs: graphene quantum dots; H_2_O_2_: Hydrogen peroxide; HA: hyaluronic acid; HCR: hybridization chain reaction; HQ: hydroquinone; KRAS: Kirsten Rat Sarcoma; LOD: the limit of detection; MCC: Mn_3_[Co(CN)_6_]_2_; Me-TTFTB: tetrathiafulvalene tetramethylbenzoate; MIL: Matériaux Institut Lavoisier; miRNA: microRNA; MOFs: Metal-organic frameworks; MUC 1: Mucin 1; N-CDs: N-doped carbon dots; N-GQDs: nitrogen-doped graphene quantum dots; NWs: nanowires; OEG: oligo-ethylene glycol; OPD: o-phenylenediamine; PL: photoluminescence; POMs: Polyoxometalates; Pt-NPs: platinum nanoparticles; PVP: poly-vinylpyrrolidone; RhB: Rhodamine B; SERS: surface-enhanced Raman scattering; SPCEs: screen-printed carbon electrodes; SPR: surface plasmon resonance; ssDNA: single-stranded DNA; T: thymine; TBTA: Tris-(benzyltriazolylmethyl) amine; tHCR: triplex-hybridization chain reaction; TMB: 3,3’,5,5’-tetramethylbenzidine; TMV: tobacco mosaic virus; TSDR: toehold strand displacement reaction; TTFTB: tetrathiafulvalene tetrabenzoate.
